# *Aspidodera* sp. infection in six-banded armadillos (*Euphractus sexcinctus*) from a German zoo

**DOI:** 10.1016/j.ijppaw.2025.101155

**Published:** 2025-10-31

**Authors:** Cora Delling, Magdalena Schollmeyer, Florian Hansmann, David Pruß, Nadine Barownick, Ronald Schmäschke

**Affiliations:** a Institute of Parasitology, Faculty of Veterinary Medicine, Leipzig University, An den Tierkliniken 35, 04103, Leipzig, Germany; b Institute of Veterinary Pathology, Faculty of Veterinary Medicine, Leipzig University, An Den Tierkliniken 33, 04103, Leipzig, Germany; c Zoological Garden Hof, Plauener Straße 40, 95028, Hof, Germany

**Keywords:** Heterakoidea, Aspidoderidae, Wildlife, Zoological garden

## Abstract

The nematodes *Aspidodera* spp. are occurring in different mammals of the southern Nearctic and Neotropical region. Six-banded armadillos (*Euphractus sexcinctus*) are mainly found in South America and act as suitable hosts for different *Aspidodera* species. Here, a case of an *Aspidodera* sp. infection in six-banded armadillos from a German zoo is described. A 17-year-old male six-banded armadillo found in poor body condition was euthanized for ethical reasons. The parasitological examination of the gastro-intestinale tract revealed an infection with nematodes of the superfamily Heterakoidea. Sequence analysis targeting the 18S rRNA confirmed high identities with isolates of *Aspidodera* sp. (accession number: EF180070; 100 %) and *Aspidodera raillieti* (accession number: KX954128; 99.86 %). Analysis of faecal samples of the remaining female armadillo led to similar results. This case description broadens the knowledge about parasitic infections in armadillos from zoological gardens in Europe.

## Highlights

• Heterakoidea eggs were found in the intestinal content and faecal samples of six-banded armadillos.
• Targeting the 18S rRNA gene very high identities with isolates of *Aspidodera* sp. and *A. raillieti* were detected.
• First description of *Aspidodera* sp. infection in six-banded armadillos from a German zoo.

## Graphical abstract

**Figure:**
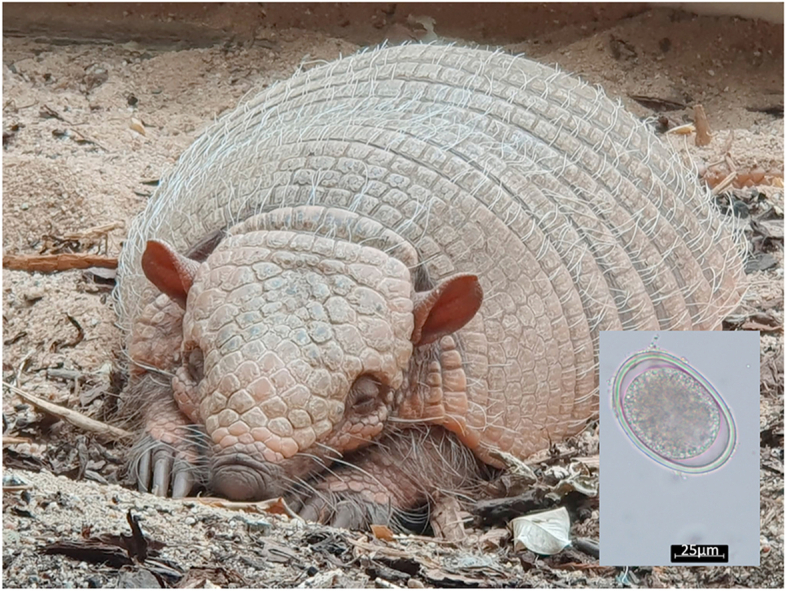

## 1 Introduction

Nematodes of the family Aspidoderidae, Freitas, 1956, are commonly occurring in the southern Nearctic and the entire Neotropical region and parasitize in the large intestine of didelphiomorphs, hystriocognath, and sigmodontine rodents as well as xenarthrans (Jiménez-Ruiz et al., 2006). For morphological species identification of *Aspidodera*, structures near the anterior end of these nematodes, namely “hoods” and “cordons” representing cuticle covered grooves, are typically used, as well as, in case of male individuals, the shape and length of spicules, the shape of the spinneret, the number of caudal papillae, and the precloacal succer surrounded by a cuticular rim in many taxa (Chagas-Moutinho et al., 2007; Jiménez-Ruiz et al., 2006). Nematodes of the species *A. raillieti* have a monoxenous life cycle and they are found in a wide range of different host species e.g., *Didelphis albiventris*, *Nectomys squamipes*, and *Philander quica* (Varella et al., 2022). The clinical importance of these parasites is quiet unknown since infection is described almost exclusively in wildlife (Superina, 2000).

The natural habitats of six-banded armadillos are dry and parts of wet savannahs within a wide geographical distribution in South America, and since they are omnivores, their diet includes insects, small vertebrates, plant material and carrion (Redford and Wetzel, 1985). Nematodes from the superfamily Heterakoidea which were described in six-banded armadillos so far are *Aspidodera binansata*, *A. fasciata, A. scoleciformis,* and *Lauroia bolivari* (Hoppe et al., 2009; Jiménez-Ruiz and Gardner, 2003). While little information is available about the occurrence of those nematodes in armadillos from zoos, especially in Europe, helminths of the families Trichostrongylidae and Strongyloididae as well as coccidia seem to be diagnosed frequently in animals under human care (Superina, 2000).

Herein, a case of *Aspidodera* sp. infection in six-banded armadillos (*Euphractus sexcinctus*) from a German zoo is described.

## 2 Material and methods

### 2.1 Clinical presentation, patient history, and housing conditions

A 17-year-old male six-banded armadillo (*Euphractus sexcinctus*) from the zoological garden Hof was suddenly found in poor general condition without showing any clinical signs before. Since the conducted therapeutic attempt did not improve the health status, the animal was euthanized for ethical reasons.

The armadillo was born in a zoological garden in Denmark, and was brought to a zoological garden in Germany at the age of seven months. In 2023, the animal moved to the zoological garden Hof, Germany. Before moving, the animal's faecal samples were examined and found negative for bacterial and parasitological pathogens. Within the first months in Hof, the armadillo showed no weight increase despite an abundant food supply. Therefore, an additional parasitological examination was conducted by an external company, and eggs of *Ascaridia* sp. were identified. The animal was treated with fenbendazole, and afterwards, the armadillo showed normal weight gain. The other six-banded armadillo living in the zoological garden Hof was a 13-year-old female, which was born in a German zoo 2011 and moved to the zoo Hof later on in 2023.

The armadillos' enclosure in the zoological garden Hof is tempered at 24 °C constantly, and sand as well as woodchips are used as flooring material. The animals were fed with vegetables, insects, eggs, curd, and chicks. The armadillos were kept together during the mating season, outside of this period, housing was performed individually or in groups depending on the animals’ temper.

### 2.2 Pathological examination

Macroscopic examination of the six-banded armadillo was performed. For histopathology a collection of representative tissue samples was taken. Tissue samples were fixed in 4 % neutral buffered formaldehyde, trimmed and embedded in paraffin wax. Slices were stained with hematoxylin and eosin for subsequent microscopic examination.

### 2.3 Parasitological examination

To identify adult parasitic stages, the gastro-intestinal tract was examined macroscopically first. Further on, a combined sedimentation-flotation technique was conducted for examining the intestinal content as described by Schmäschke (2014). In brief, the sample was sieved to eliminate larger particles. After 30 min of sedimentation, the supernatant was decanted and 1 ml of the sediment was floated with sodium nitrate for 5 min using a centrifuge (2000 rpm). Additionally, DNA was isolated from two adult helminths found in the intestine by using the QIAmp® DNA Mini Kit (QUIAGEN, Hilden, Germany) following the manufacturer's instructions. For sequence analysis, a PCR was performed targeting the partial region spanning 18S rRNA using the primers Asp-18SF (5′CGTTCCGTCGGCGGTAAATATG3′) and R136 (5′TGATCCTTCTGCAGGTTCACCTAC3′) as described elsewhere (Santos et al., 2019; Nadler et al., 2007). The PCR conditions for each reaction were as following: 0.5 μl of each primer, 0.1 U of DreamTaq DNA polymerase (ThermoFisher Scientific, Dreieich, Germany), 2.5 μl of DreamTaq™ Green Buffer (10x; ThermoFisher Scientific, Dreieich, Germany), and 0.8 μl of dNTP. Three microliters of the DNA sample were used and DNA-free, nuclease-free water was added to achieve a final volume of 25 μl. The following thermal protocol was used: 95 °C for 5 min; 95 °C for 30 s, 56 °C for 30 s, 72 °C for 1 min (35x) followed by 72 °C for 7 min. After conduction of gel electrophoresis, ethidium bromide was used for gel staining and bands were visualized by UV-light. The PCR products were purified by using the PCR Purification Kit (Jena Bioscience GmbH, Jena, Germany). Sequencing was performed in both directions by a commercial company (Microsynth Seqlab, Göttingen, Germany). The sequences were aligned, compared with those available in the GenBank database by BLASTn analysis (http://blast.ncbi.nlm.nih.gov/Blast.cgi), and phylogenetic analysis was performed using the Maximum Likelihood method and Kimura 2-parameter model in MEGA version X (Kumar et al., 2018; Felsenstein, 1985; Kimura, 1980). The sequence obtained in this study was submitted to GenBank (SUB15691109).

Additionally, a parasitological examination of the faeces from the remaining female armadillo was performed. Therefore, the combined sedimentation-flotation technique was conducted. Furthermore, DNA was extracted using the QIAmp® Fast DNA Stool Mini Kit (QUIAGEN, Hilden, Germany). PCR and sequence analysis were performed as described above.

## 3 Results and discussion

### 3.1 Pathological investigation

At gross examination the animal was showed a poor body condition, with absence of subcutaneous and visceral adipose tissue. The oral cavity was devoid of any teeth.

Histopathology revealed a mild suppurative and lymphoplasmahistiocytic rhinitis. This finding was not considered to be the cause of the poor body condition and the clinical deterioration.

Furthermore, a mild bile duct hyperplasia in the liver and storage of a mild amount of lipofuscin in the cytoplasm of neurons were observed. These findings were interpreted as morphological changes of minor or no clinical significance and could be associated with the age of the animal. In the gastrointestinal tract no lesions were detected.

### 3.2 Parasitological examination

**Fig. 1:**
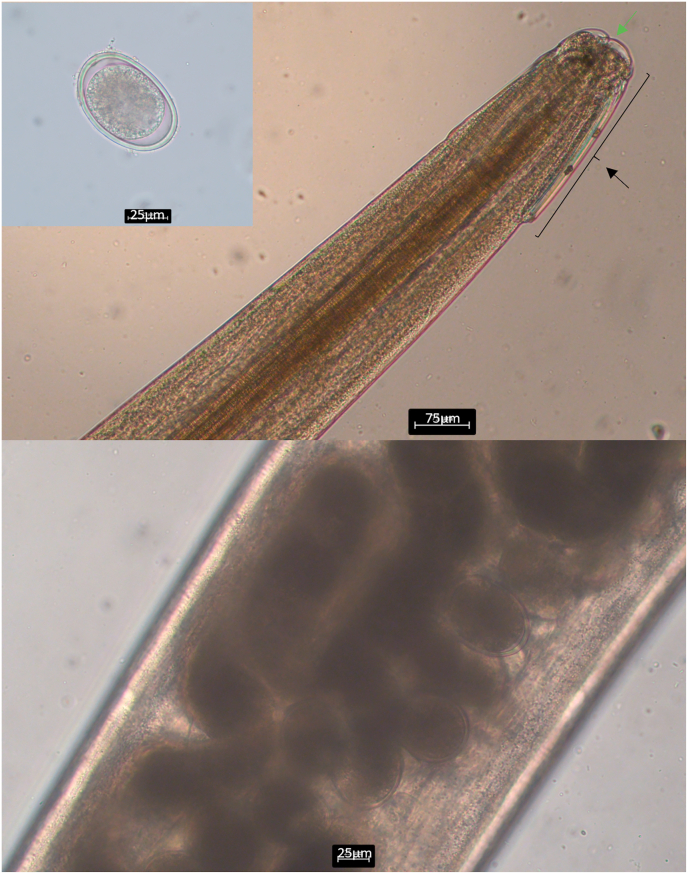
Light microscopy of parasitic stages found in the six-banded armadillos (*Euphractus sexcinctus*) a) Egg of the superfamily Heterakoidea; b) Anterior end of a female *Aspidodera* sp. with hood (black arrow), lips (green arrow), and cordons; c) Uterus with heterakoid eggs.

**Fig. 2:**
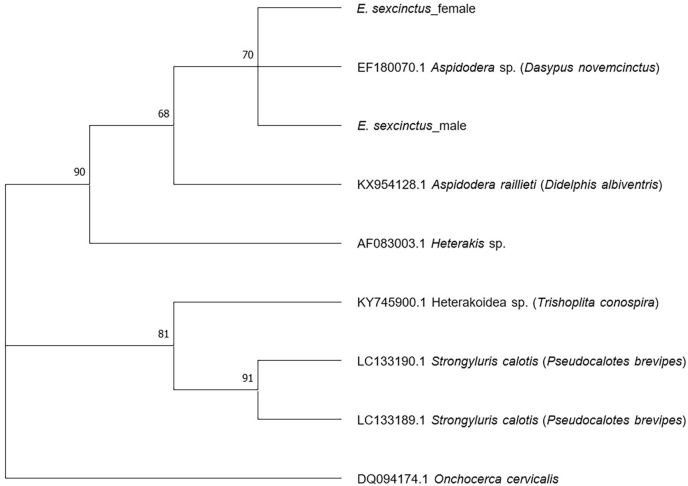
Phylogenetic analysis of the sequences obtained in this study (*E. sexcinctus*_male/female) and exemplary sequences from GenBank using Maximum Likelihood method and Kimura 2-parameter model. Only branches with support values greater than 50 % are shown. A sequence of *Onchocerca cervicalis* (accession number: DQ094174.1) was used as outgroup.

Examining the male armadillo's intestine, a few adult parasitic stages and eggs of the superfamily Heterakoidea (Railiet and Henry, 1912) were detected (Fig. 1). Targeting the 18S rRNA gene, sequence analysis revealed very high identities with isolates of *Aspidodera* sp. (accession number: EF180070; 100 %; query cover: 99 %) and *A*. *raillieti* (accession number: KX954128; 99.86 %; query cover: 99 %). Analysing the faecal samples of the remaining female armadillo, eggs of the superfamily Heterakoidea were also found, and sequence analysis generated the same results as those of the male individual. Phylogenetic analysis showed the clustering of the isolates found in this study and the isolate of *Aspidodera* sp. (accession number: EF180070) which was found in a nine-banded armadillo (*Dasypus novemcinctus*) from Costa Rica (Fig. 2). Species of Aspidoderid nematodes described before in nine-banded armadillos from the Americas are *A. binansata*, *A. fasciata*, *A. scoleciformis*, *A. sogandaresi*, *A. vazi, Lauroia bolivari*, and *L. trinidadensis* (Santos et al. 1990, 2019; Jiménez et al., 2012; Hoppe and Nascimento, 2007).

Interestingly, the isolates of the six-banded armadillos showed 99.86 % identity with *A. raillieti* which is found regularly in the common opossum (*Didelphis marsupialis*), the Andean white-eared opossum (*Didelphis pernigra*), and the Big-eared opossum (*Didelphis aurita*) from the United States and South America (Jiménez et al., 2012; Chagas-Moutinho et al., 2007). There have been no reports of *A. raillieti* in armadillos so far, although they cover a wide host range occurring in the Americas from Argentina to southern Illinois. Despite their wide distribution, infection seems to be restricted to a defined set of mammals (Jiménez et al., 2012). Distribution and dimension of specificity of several *Aspidodera* species in Dasypodidae and Chlamyphoridae remain to be clarified (Jiménez et al., 2012).

Infections with nematodes of the superfamily Heterakoidea are usually reported from hunted or road-killed armadillos from the Americas, and information about infected animals under human care is lacking, although these animals are kept frequently in zoological gardens (Santos et al., 2019; Jiménez-Ruiz et al., 2006). According to our findings, *Aspidodera* spp. appear to occur also in Europe, nevertheless, the dimension of their distribution in armadillos from zoological gardens is unknown. Furthermore, the impact of infection with these helminths on animals' health is difficult to assess so far. In this case, the nematodes appeared to have no or little effect on the clinical presentation of the armadillo, whereby the infection rate was relatively low. Therefore, further investigations could help to broaden the knowledge of the parasites’ influence on the host and to estimate the distribution of Aspidoderid nematodes in Europe. To the best of our knowledge, this is the first report of *Aspidodera* sp. infection in six-banded armadillos from Germany.

## CRediT authorship contribution statement

**Cora Delling:** Funding acquisition, Investigation, Writing – original draft. **Magdalena Schollmeyer:** Investigation, Writing – review & editing. **Florian Hansmann:** Investigation, Writing – review & editing. **David Pruß:** Investigation, Writing – review & editing. **Nadine Barownick:** Investigation. **Ronald Schmäschke:** Supervision, Writing – review & editing.

## Funding

Supported by the Open Access Publishing Fund of 10.13039/501100008678Leipzig University.

## Declaration of interest

All authors concur with the submission and the material submitted is not under consideration for publication elsewhere. The authors declare there is no conflict of competing, financial or non-financial interests.
